# VMXi: a fully automated, fully remote, high-flux *in situ* macromolecular crystallography beamline

**DOI:** 10.1107/S1600577518015114

**Published:** 2019-01-01

**Authors:** Juan Sanchez-Weatherby, James Sandy, Halina Mikolajek, Carina M. C. Lobley, Marco Mazzorana, Jon Kelly, Geoff Preece, Rich Littlewood, Thomas L.-M. Sørensen

**Affiliations:** a Diamond Light Source, Harwell Science and Innovation Campus, Chilton, Didcot, Oxfordshire OX11 0DE, UK; b Department of Molecular Biology and Genetics, Aarhus University, Gustav Wieds Vej 10, 8000 Aarhus C, Denmark

**Keywords:** room-temperature data collection, *in situ* diffraction experiments, automated beamline operation, serial crystallography

## Abstract

VMXi is a new high-flux microfocus macromolecular crystallography beamline at Diamond Light Source. The beamline, dedicated to fully automated and fully remote data collection of macromolecular crystals *in situ*, allows rapid screening of hundreds of crystallization plates from multiple user groups. Its main purpose is to give fast feedback at the complex stages of crystallization and crystal optimization, but it also enables data collection of small and delicate samples that are particularly difficult to harvest using conventional cryo-methods, crystals grown in the lipidic cubic phase, and allows for multi-crystal data collections in drug discovery programs. The beamline is equipped with two monochromators: one with a narrow band-pass and fine energy resolution (optimal for regular oscillation experiments), and one with a wide band-pass and a high photon flux (optimal for fast screening). The beamline has a state-of-the-art detector and custom goniometry that allows fast data collection. This paper describes the beamline design, current status and future plans.

## Abstract 2

VMXi, a new tunable beamline dedicated to fully automatic screening and data collection from crystals *in situ*, is reported.

## 1. Introduction   

As we are looking for answers to ever more complex scientific questions in structural biology, projects are becoming increasingly challenging. Many of the samples we are studying are difficult to express and purify, target proteins are likely to be flexible, crystallization from miniscule amounts of sample is highly demanding, and, when crystals are obtained, they are often small and diffract poorly.

Here we introduce the VMXi beamline at Diamond Light Source (DLS), a new microfocus macromolecular crystallography (MX) beamline dedicated to fully automatic screening and data collection from crystals *in situ*. The VMXi beamline addresses a number of related current needs in MX: (1) identifying diffraction-quality crystals for biological systems that are difficult and expensive to crystallize (*e.g.* membrane proteins; large complexes), (2) obtaining diffraction datasets for the biological systems of which crystals are routinely resistant to cryogenic harvesting (*e.g.* large complexes; viruses; membrane proteins), and (3) characterizing protein–ligand interactions rapidly by collecting large numbers of datasets from similar crystals with different compounds added (fragment screening and industrial drug development).

Crystallization is often a very long and iterative process where initial ‘promising’ conditions are optimized until crystals suitable for the diffraction experiments are obtained. Crystallization optimization is frequently undertaken without X-ray diffraction information. Crystals are more often than not optimized based on their appearance, due to the difficulty of cryo-cooling and shipping every potential sample to the synchrotron. Standard procedures when preparing crystals for cryo-cooled data collection involve the manual manipulation of individual crystals, soaking them in cryo-protectants and subsequent cryo-cooling. Inevitably, the mechanical and osmotic stress that occurs during these steps may significantly affect the diffraction capabilities of the crystal.

The *in situ* diffraction approach that VMXi is providing means that (1) experiments can be carried out without any manipulation of individual crystals, thus preserving the crystal integrity, (2) immediate feedback on the diffraction, crystal quality, unit-cell parameters and space group, even in the case of micro-crystals, and (3) the method can be fully automated with high reliability.

The ability to rapidly characterize the diffraction properties of crystals using *in situ* diffraction provides a particular competitive edge for the analysis of unstable large macromolecular complexes, membrane proteins, and for the assessment of initial micro-crystal hits. At VMXi it is possible to test whether your ‘micro crystal’ hits are protein and whether they diffract, providing essential and immediate feedback, so that either crystals can be further optimized or, equally importantly, false positives can be rapidly discarded, saving both time and money for the researcher. This is particularly valuable in the case of membrane protein crystals grown in the lipidic cubic phase where the harvesting step is extremely problematic (Caffrey, 2003 ▸).

The development of VMXi has benefitted from recent developments in automation and *in situ* data collection at other beamlines around the world and from significant technological developments. As synchrotron beams have become brighter and better focused, and detector readout has become faster and more sensitive (Casanas *et al.*, 2016 ▸; Rajendran *et al.*, 2011 ▸; Aishima *et al.*, 2010 ▸), we have seen a surge in multi-crystal room-temperature data collection (Broecker *et al.*, 2018 ▸; Aller *et al.*, 2015 ▸). Crystal radiation damage can be outrun due to the increased dose-rate (Owen *et al.*, 2012 ▸; Schubert *et al.*, 2016 ▸; Chapman *et al.*, 2014 ▸) making it possible to collect small wedges of data at room temperature. Data collection and data processing tools have evolved around these new developments and it is now standard practice using *in situ* data collection to be able to obtain full structural information from partial datasets on multiple samples (Foadi *et al.*, 2013 ▸; Zander *et al.*, 2015 ▸; Santoni *et al.*, 2017 ▸; Stellato *et al.*, 2014 ▸). It is a huge step forward for many challenging projects to be able to analyse samples *in situ*, bypassing the additional sample manipulation associated with cryo-cooling methods.

*In situ* crystallography is possible using a laboratory-based X-ray source (PlateMate, Rigaku) (Hargreaves, 2012 ▸), but is now also offered routinely at a number of synchrotron facilities around the world, including the DLS beamlines I03 and I24 (Aller *et al.*, 2015 ▸; Axford *et al.*, 2012 ▸; Materlik *et al.*, 2015 ▸; Allan *et al.*, 2015 ▸), the SLS beamline X06DA (PXIII) (Bingel-Erlenmeyer *et al.*, 2011 ▸), the ESRF beamlines BM30 and BM14 (le Maire *et al.*, 2011 ▸), the APS beamline GM/CA (Broecker *et al.*, 2016 ▸) and the PETRA beamline P14 (DESY, Hamburg, Germany).

### 1.1. Beamline overview   

The VMXi beamline replaces the tunable MX beamline I02 (Allan *et al.*, 2015 ▸; Grimes *et al.*, 2018 ▸) which ended operations in August 2016. The original beamline layout has been extended by moving the sample position downstream and adding an additional experimental hutch and sample storage area. This configuration has provided a completely new sample area and allowed I02 to continue user operations during the build and installation of the VMXi beamline. The optical path has been reconfigured by repurposing components from the original I02 layout while extending specifications by adding a multilayer monochromator, a secondary source point and a pair of microfocus bimorph mirrors. In addition, the beamline is equipped with a new state-of-the-art detector and custom goniometry allowing very fast and precise data collections.

### 1.2. Operational overview   

The VMXi beamline introduces a fundamentally different approach to how scientists use and interact with a beamline. The extensive level of automation introduced means that users no longer operate the beamline directly and have no specific beam time slot allocated. Instead, crystallization plates are stored at the beamline and users evaluate the crystallization results via a bespoke GUI in SynchWeb (Fisher *et al.*, 2015 ▸). Data collection requests are detailed and submitted from the web interface and all requested beamline operations are carried out in a fully automated fashion. Data are processed automatically and the users are only notified when new data are available for review. This operating principle ensures optimal use of the available X-rays by decoupling the decision making of which data to collect and how to collect the data from beam time. It is matched to the unpredictable nature of the crystallization process where beam access is required once crystals appear rather than the other way around.

## 2. X-ray beam path   

**Figure 1:**
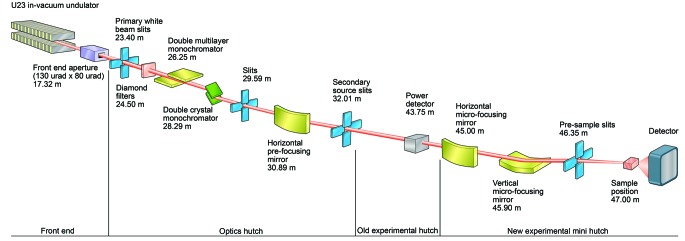
Schematic of the beamline outline detailing the main optical components along the beam path from source to sample. Figure not drawn to scale but distances to the source in metres for each component are marked.

The VMXi X-ray beam path stretches 47 m from the source at the 2 m *in vacuum* U23 undulator (located in the centre of the 02 straight section of the DLS storage ring) to the sample position as outlined in Fig. 1 ▸ and detailed in Table 1 ▸.

### 2.1. Monochromators   

The beamline has a unique configuration with two separate monochromators which can be used depending on the experimental requirements: the original double-crystal monochromator (DCM) for narrow band-pass beam with lower flux and the new double-multilayer monochromator (DMM) for broader band-pass beam with higher flux. The monochromators are designed to enable beamline operation in either DCM or DMM configuration.

The DCM is an in-house upgrade of an original FMB Oxford design (Allan *et al.*, 2015 ▸). The cryo-cooled Si(111) fixed-exit monochromator delivers a full energy range from 5 to 25 keV with a band-pass (Δ*E*/*E*) of <10^−4^ giving a flux at 13 keV of >2 × 10^12^ photons s^−1^ at the sample position. The DCM is the preferred standard option for MX data collection typically used for collecting oscillation data on single crystals and for measuring X-ray fluorescence for phasing purposes or low-dose X-ray scanning for locating small crystals.

The DMM is an in-house design described elsewhere (manuscript in preparation). This monochromator has two separate silicon crystals with two distinct multilayer coatings stripes each. The multilayers coatings are built of alternate layers of ruthenium (Ru) and boron carbide (B_4_C) with layer thicknesses of 2.0 nm and 2.4 nm for the two stripes, respectively (Rigaku). By using either of the stripes, the DMM delivers beam across the energy range 10–25 keV with a band-pass (Δ*E*/*E*) of <5 × 10^−2^ giving a flux at 13 keV of >10^14^ photons s^−1^ at the sample position. The DMM is primarily used when a higher flux and broader band-pass is advantageous such as for raster scanning across large regions of a crystallization drop. The higher flux increases the speed at which large area scans can be completed, while the broader band-pass also improves the likelihood of correct cell parameters being assigned from still images by generating an effect akin to a small oscillation (Nave, 2014 ▸). Initial tests (see the *Results* section[Sec sec6]) suggest that, despite severe radiation damage, single-crystal oscillation data collections using the DMM can be processed with standard software and produce good quality data. We expect that, as the crystal lifetime is better defined and optimized, the DMM will also be routinely used for oscillation data collection.

We have also installed a low band-pass diamond filter upstream from the DMM to prevent low-energy radiation from the storage ring being reflected off the multilayer surfaces due to the very shallow operating angles used. This could cause low-energy contamination of diffraction as well as significant radiation damage to hardware further along the beam path. The specification and operational ranges for these diamond filters have been summarized in Table 1 ▸.

### 2.2. Focusing optics   

#### 2.2.1. Horizontal pre-focusing mirror and the secondary source   

To achieve a micro-focused beam at the sample position, we have introduced a secondary source point in the horizontal plane at 32 m by repurposing the horizontal-focusing bimorph mirror (HFM) previously used on the I02 beamline (Allan *et al.*, 2015 ▸; Grimes *et al.*, 2018 ▸). The mirror angle of 2.7 mrad has been maintained but the bimorph voltages were increased to adjust the curvature to move the focus from the 40 m it had been previously to the new secondary source point at 32 m. This enables us to obtain a horizontal beam size at the sample position of 6 µm (FWHM) which can be further reduced down to 1–2 µm by trimming the beam at the secondary source point.

The new secondary source combines movable slits, a beam-position monitor, a diamond scintillator screen and a camera imaging the beam to facilitate optimization of the secondary source beam properties.

#### 2.2.2. Microfocusing mirrors   

**Figure 2:**
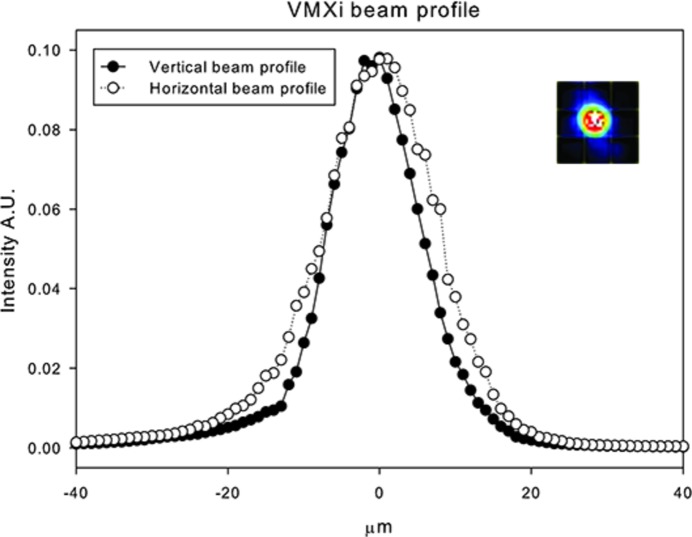
Current beam profile measured on a PIN diode while scanning a 10 µm-diameter aperture across the beam vertically and horizontally at the sample position. The insert shows an image of the attenuated beam on a YAG scintillator. The image depicts a 30 µm × 30 µm area and the beam is approximately 10 µm across. Further optimization of the beam focus is ongoing.

The beamline is equipped with a horizontal microfocus mirror (HMFM) and a vertical microfocus mirror (VMFM). The microfocus mirrors are located at the front of the new VMXi mini-hutch, 2 m and 1 m from the sample, respectively. To achieve optimal focused beam given the very tight location restrictions, both mirrors share a single vacuum vessel. The microfocus mirrors are super-polished SiO_2_ substrate with 16 side-mounted piezo elements for shape adjustment (Alcock *et al.*, 2015 ▸). The mirrors are polished to an elliptical shape to enable optimal focusing and defocusing and operate at 3 mrad. The mirrors have three different 12 mm-wide reflective areas to provide optimal reflectivity and harmonic rejection across the full energy range: one uncoated area used below 10 keV, a rhodium-coated area used between 10 and 18 keV, and finally a platinum-coated area providing optimal reflectivity at energies above 18 keV. Beam size and focal point can be modified by adjusting the voltage supplied to the 16 piezo actuators from the high-voltage power supply (CAEN ELS SRL). To achieve the focal range of 0 to 300 mm downstream from the sample and focal sizes ranging from 5 µm × 5 µm to 30 µm × 30 µm, the mirror voltages are operated between −1500 V and +1500 V (Fig. 2 ▸).

The mirror vessel also includes a ‘dissector’ located upstream of the mirrors. The dissector has dual functionality and is designed to help with mirror optimization and also to act as a beam monitoring aperture. The small tungsten apertures of 10 and 20 µm in the horizontal and vertical planes are used to optimize the beam focus, using pencil-beam scans required for piezo voltage optimization (Sutter *et al.*, 2012 ▸). A third aperture in each dissector (3 mm in the horizontal and 1.2 mm in the vertical) is used to trim the incoming beam to match the optical aperture of the mirrors. In addition, these beam-trimming apertures allow drain current measurement providing beam position feedback for the upstream optics.

### 2.3. Beam conditioning   

As the beam converges towards the sample position a final set of elements are used to condition and fine-tune the beam. The beam passes through a set of slits, which are set to the nominal beam size to eliminate any unwanted scatter produced upstream. These slits can also be used to create a smaller aperture to reduce beam divergence. This will generate sharper Bragg reflections at the detector to improve the signal-to-noise ratio and resolve Bragg reflections for larger unit cells.

**Figure 3:**
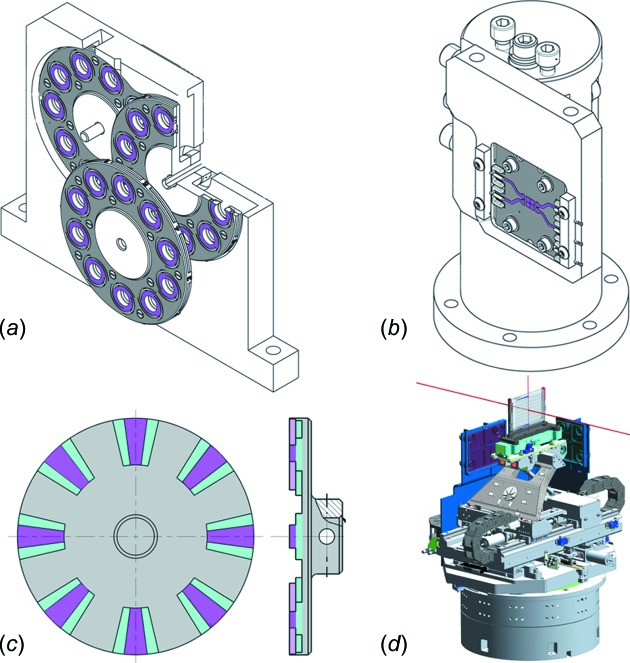
(*a*) The attenuator wheel assembly. The foils are held inside each of the inserts and the beam passes through the point where all three wheels intersect. (*b*) CIVIDEC XBPM mount. The four active pads used for monitoring beam intensity and position are coloured pink. (*c*) Sample shutter showing the stepped arrangement of the blades that allow for altering patterns of opening and closing. Blue is 50/50 and pink is 75/25. (*d*) Goniometer assembly showing the air-bearing, the scissor-like *x–y* translation stages, the *z* motion, the mirror interferometers and the tilt mechanism.

The beam can be attenuated by using aluminium and silver foils of various thickness (from 10 µm to 1.5 mm Al and 0.1 mm to 0.8 mm Ag). The filters are mounted on three motorized wheels each holding sets of ten foils [Fig. 3(*a*) ▸] that can be placed in the beam path giving rise to 1000 individual filter combinations, giving a fine range of beam attenuation across the energy range 10–25 keV for both DCM and DMM beams. The beam position is carefully monitored using a four-quadrant diamond-based beam-position monitor (XBPM) (CIVIDEC Instrumentation GmbH) [Fig. 3(*b*) ▸]. Attenuator filters and the XBPM are housed in a helium-filled environment to prevent oxidation and to aid motor cooling. The oxygen level in the chamber is monitored by using an InPro oxygen sensor (Mettler Toledo).

Having passed through the XBPM, the beam reaches the rotary shutter. The shutter is a rotating disk positioned perpendicular to the beam axis and is driven by an EC-type motor (Maxon Motors) [Fig. 3(*c*) ▸]. The design of the shutter allows it to be used in a stop–start mode as a very fast conventional shutter with an opening time from fully closed to fully opened of only 50 µs. It is also possible to operate the shutter in continuous mode where the disk rotates and the beam is ‘chopped’ eight times per revolution of the wheel. The motor is able to reach a speed of 5625 rev min^−1^ enabling 750 Hz data collection frequency, matching the frame rate capabilities of the beamline detector. The blades on the chopper disk have a tiered design that allows the ratio of open/close time to be varied between 50/50 and re-focusing 70/25 in the current configuration by translating the chopper across the beam. To minimize background, the ‘chopper’ mode can be synchronized with the detector to allow a sample to only be exposed to X-rays for a fraction of each collected frame rather than in a continuous exposure. This mode will be used when collecting serial crystallography data.

**Figure 4:**
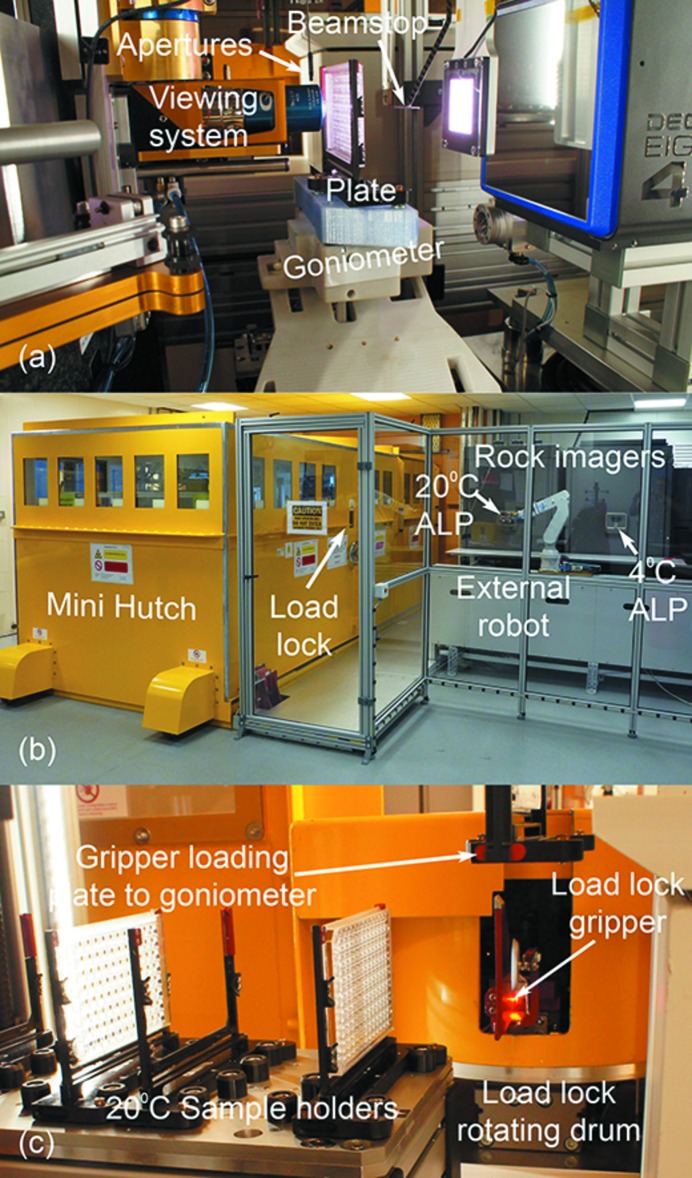
(*a*) Global view of the sample environment while a sample well is being imaged. (*b*) Overview of the VMXi experimental hutch, the Rock Imager storage units and the six-axis robot. (*c*) View of the 293 K sample local storage section. Empty and loaded sample holders are shown as well as a loaded sample holder in transit to the goniometer. The internal drum of the rotation load lock that allows samples to be loaded and unloaded through the closed radiation enclosure is also shown.

The final element before the sample is a set of three platinum–iridium apertures, 10, 50 or 200 µm in diameter (Agar Scientific) [Fig. 4(*a*) ▸]. The apertures remove any unwanted scatter, helping to reduce background on the detector.

The beamstop is the final component in the beam path after the sample position before the detector [Fig. 4(*a*) ▸]. In addition to preventing damage to the detector, the beamstop is also crucial for maximizing the quality of the diffraction data. The design reduces unwanted background while still allowing the collection of good quality data at low resolution. The current beamstop is a set of interlinked tubes as first described by Meents *et al.* (2017 ▸). In the VMXi configuration, the beamstop is made of three interlocked platinum tubes (400, 600 and 950 µm outer diameter) with the large tube plugged with gold wire and lead. It can be positioned 15 mm behind the sample, and at this distance the beamstop allows low-resolution reflections of approximately 93, 71 and 37 Å at energies of 10, 13 and 25 keV, respectively, to be recorded.

### 2.4. Detector   

At the end of the beam path is an EigerX 4M detector (Dectris) (Johnson *et al.*, 2012 ▸; Casanas *et al.*, 2016 ▸; Tinti *et al.*, 2017 ▸) [Table 2 ▸ and Fig. 4(*a*) ▸]. This detector has an active area of 155.2 mm × 162.5 mm (W × H) with a pixel size of 75 µm × 75 µm. It is able to collect data continuously at 500 Hz and in short bursts of up to 30 s at 750 Hz. When operating at full flux the limiting factor is the photon count rate of 2.4 MHz per pixel as well as the bit depth of 12 bits. The detector performance will improve when VMXi starts operation with a new Eiger2X 4M detector at the beginning of 2019. This detector will have a significantly improved photon count rate, counter bit depth and effectively have no readout time. Further improvements will await the availability of a suitable charge integrating detector, which promises a tenfold increase in photon count rate.

### 2.5. Beam monitoring and feedback   

For VMXi, all aspects of beam delivery and diagnosis are automated and three elements along the beam path provide feedback to maintain stable beam at the sample position. Firstly, drain current on the primary slits (located upstream from the monochromators) is used to monitor changes to the incoming beam from the storage ring. Secondly, changes in drain current on the dissector blades before the microfocus mirrors are used to adjust the pitch of the pre-focusing HFM. Finally, an X-ray beam-position monitor (CIVIDEC) upstream from the sample shutter is used to ensure that the beam is stable at the sample position by adjusting the horizontal and vertical pitch of the microfocus HMFM and VMFM. Monitoring currently runs at 10 Hz and feedback at 0.2 Hz but is disabled during data collection.

## 3. Sample path   

To make unattended operations possible, VMXi has a fully automated sample path that allows for the transfer of sample plates from storage outside the experimental hutch to the goniometer inside the hutch, and back [Fig. 4(*b*) ▸]. By storing the samples outside the radiation-shielded enclosure, we have maintained easy access to stored samples even while the beamline is operating. Furthermore, this set-up makes future upgrades to increase sample storage capacity straightforward, without the need of modifications to beamline radiation shielding.

The beamline is currently set up to handle SBS-format crystallization plates (ANSI/SLAS 1-2004 through ANSI/SLAS 4-2004), and with appropriate adapters the beamline can handle smaller-sized plates. The SBS format has proved longevity and our assessment is that any future formats are likely to have either the same footprint with higher density or be smaller in which case adapters to mimic the SBS footprint can be used during a transition phase while upgrading the beamline to handle a new format.

### 3.1. Sample storage   

User plates are stored on the beamline in one of two modified Rock Imager 1000 units (Formulatrix, USA) operating at 277 K and 293 K, respectively [Fig. 4(*b*) ▸]. These units store and automatically image up to 750 crystallization plates each. Additional features have been added to the VMXi Rock Imagers in collaboration with Formulatrix to allow full beamline integration.

**Figure 5:**
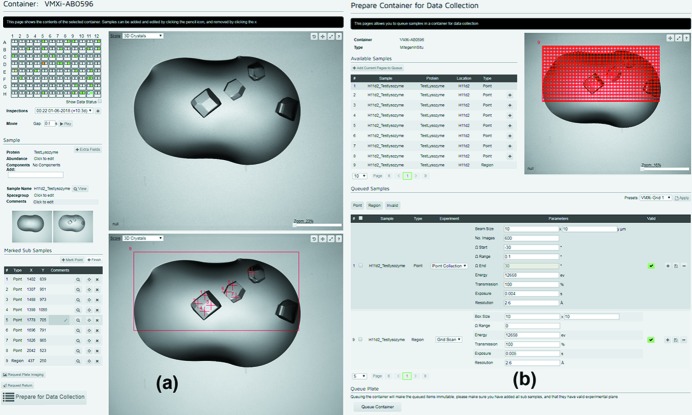
(*a*) The GUI tools in SynchWeb that allow users to review crystallization results, annotate drops, mark points and select regions for data collection. (*b*) SynchWeb view for setting data collection parameters and requesting collection of data.

The imaging modules in the Rock Imagers have been inverted to image crystallization plates through the bottom of the plates (Fig. 5). This means that crystallization drops are imaged from the same direction on and off the beamline enabling a more robust sample alignment. Furthermore, the Rock Imagers have been fitted with an automation load port (ALP) at the back of the units used for automatic transfer of plates between storage and the beamline for diffraction experiments [Fig. 4(*b*) ▸].

### 3.2. Sample transfer   

Sample transfer and data collection have been implemented, both at the software and hardware level, as separate concurrent processes so that sample loading and unloading does not affect the beamline output and *vice versa*. To achieve this, a radiation-safe transfer system (load lock) shown in Figs. 4(*b*) and 4(*c*) ▸ allows samples to be loaded in an out through the radiation enclosure while data are being collected. Furthermore, sample storage located inside the hutch allows twelve plates to be kept next to the goniometer (six at 277 K and six at 293 K), thus allowing quick exchange to the goniometer to maximize data collection time [Fig. 4(*c*) ▸].

A sample transfer starts with the crystallization plate being delivered to the ALP in the Rock Imager. Once ejected via the ALP, the plate is picked up by a robot arm (RV-2FL MELFA, Mitsubishi Electric) mounted on a track running perpendicular to the beam path and alongside the Rock Imager storage units [Fig. 4(*b*) ▸]. The robot arm delivers the plate to the load lock.

Once inside the shielding, the plate is picked up by a gantry robot (Sysmac Automation Platform, Omron) and transferred to the local storage area. Holding plates in adjustable high-precision plate holders [Figs. 3(*d*) ▸, 4(*a*) and 4(*c*) ▸] ensures repeatable positioning on the goniometer improving accuracy when collecting data. Using individual holders reduces the time required to swap between data collection at 277 K and 293 K as it prevents waiting for a common holder on the goniometer to change temperature.

The waiting plate and holder are then transferred onto the goniometer [Figs. 3(*d*) ▸ and 4(*a*) ▸]. This procedure allows an exchange time of plates of less than 20 s. To maintain the temperature of the 277 K plates and holders during data collection, the gantry robot mounts an additional cold cover over the plate, while in cold storage, before transferring the holder to the goniometer. This cold cover maintains the temperature of a plate at 277 K for up to 15 min on the goniometer.

All steps in the transfer are monitored to capture any anomalies and prevent damage to the plates or holders. Gripper sensors, barcode scanner and a proximity laser have been integrated to give the system the ability to detect and identify the status of transfer at every step of the process.

### 3.3. Goniometry   

The crucial challenge for the goniometer design has been to achieve speed and precision to enable rapid translocation to the desired drop position within the SBS format while aligning and rotating micrometre-sized crystals in the beam (Table 2 ▸). The goniometer design enables data collection while oscillating the sample (omega scans) and data collection while moving the sample through the beam (area scans), and combinations of the two. The novel goniometer design [Fig. 3(*d*) ▸] has a rotation axis (RT300L, Nelson Air) at the base aligned to the focal point of the beam. On this air bearing, we have placed the sample translation axes where a scissor-like design with two horizontal translations at the base (EC-type, Maxon Motors) actuate on two arms creating virtual horizontal and vertical sample axes as seen in Figs. 3(*d*) ▸ and 4(*a*) ▸. This motor configuration allows for very fast motions up to 100 mm s^−1^ without bulky translation stages. Given the distance from the motor encoders to the sample and the dynamic nature of the motions we are using interferometers (Attocube) mounted at the base of the sample holder to account for any parasitic moves during data collection and make on-the-fly adjustments to correct them [Fig. 3(*d*) ▸].

### 3.4. Sample imaging   

To improve the success of matching the images marked by the users to those collected at the beamline, thus ensuring a good hit rate, the beamline is equipped with a Mag.x 125 vision system (Qioptiq) and a 6 MP Manta G609-B monochrome camera (Allied Vision) [Fig. 4(*a*) ▸]. This system allows high-resolution images (>1 µm resolution) of entire drops (2 mm by 2 mm field of view) to be captured as shown in Fig. 5(*a*). The system also includes a second Optem FUSION lens (Qioptiq) with a 0.6 mm hole drilled through the centre, allowing the X-ray beam to pass through the vision system. This second lens is used for beam commissioning (Fig. 2 ▸). The lenses, which are co-axial to the beam, can be brought into position independently, and are replaced by a helium-filled tube to reduce air scatter when X-ray data are being collected (Fig. 4 ▸). Samples are illuminated using a LED RGB backlight (PHLOX).

## 4. Control software   

The beamline control software operates at three levels. An EPICS interface controls beamline hardware (EPICS, 1994 ▸) at the lowest level, and provides an interface that the higher-level software (*GDA*; GDA, 2011 ▸) has access to. *GDA* synchronizes all individual actions required to operate the beamline. Finally, users interact with the VMXi beamline solely through a bespoke GUI in SynchWeb (Fisher *et al.*, 2015 ▸) that provides the third level of software control. The key difference in the way VMXi operates, compared with other DLS beamlines, is that users never directly control the beamline. The user input is written as configuration settings to the ISPyB database (Delageniere *et al.*, 2011 ▸) and these parameters are used by *GDA* to action data collection.

Beamline autonomy is provided by *GDA* running two separate control loops. One, sample handling, is solely dedicated to loading and unloading samples to and from the beamline. The second control loop, data collection, is responsible for all of the routines needed from the point a sample is loaded on to the goniometer to the point where all data are collected and the plate is ready for unloading. To provide robustness to the system, both processes are separate and only linked via the status of the sample on the goniometer (Sharpe, 2018 ▸).

Complex routines, like image matching and sample prioritizing, are plugged in as external resources. This allows for each of the elements in the process to be optimized separately with minimal dependencies and permits expanding functionality as required with minimal risk to operations.

Robustness and error detection are critical for a fully automated beamline. To prevent clashes during operation, a range of hardware such as limit switches, cameras, sensors and lasers are used to confirm that hardware and samples are in the correct location prior to any actions. Error analysis and management is controlled by the *GDA* software coordinating all aspects of beam delivery, data collections and sample handling.

## 5. User workflow   

### 5.1. General process   

As described previously, the beamline operates automatically from data input parameters defined by the users. Users set up their crystallization experiments and review progress. Then, they mark regions or points to which data collection parameters can be assigned. Once these parameters are set up and the plate data collection has been requested, there is no further user involvement in the diffraction experiment until it comes to reviewing results and data processing.

The beamline is charged with loading and unloading the correct samples, locating the points or regions once the samples are on the beamline, preparing the beamline with the correct experimental settings, collecting the datasets and presenting the processing results.

### 5.2. User input via SynchWeb   

An overview of the user interface in SynchWeb can be seen in Fig. 5 ▸. The interface currently captures all the required sample information for safe beamline operation prior to the crystallization plate arriving at the beamline. The information gathered also includes barcode references and storage temperatures that permit the beamline staff to load the samples in their correct location. Once sample barcodes are scanned in the storage units, the barcode links the plate to the stored information in the database. The crystallization plate is then imaged automatically according to a predefined schedule and users are notified via email whenever new imaging has been completed.

As the users review their samples, they can mark points or regions on the sample images. These markers act as collection identifiers [Figs. 5(*a*) ▸ and 6(*a*) ▸]. Once the user has marked one or more points or regions, diffraction data collection can be set up. The SynchWeb interface allows the user to assign a set of experimental data collection parameters to each point or region [Fig. 5(*b*) ▸]. Once the users have supplied the required parameters the plate can be submitted for data collection. At this point, the plate joins a cohort of samples ready to be processed by the beamline for data collection.

When the beamline is ready for a new plate to be transferred to a free sample holder, the sample loading routine queries the database for the next plate to load. A selection filter is employed to determine the next plate to be selected. The selection criteria can be optimized to improve data output and to make best use of beam time available. Once a plate has been selected, it is conveyed to the beamline for data collection.

### 5.3. Sample alignment   

The alignment of a drop for data collection is a fully automated multi-step process. The first step is to position the drop by scanning it through the focal plane and look for *in focus* features. The second step is to create an extended-focus image by merging twelve images taken every 20 µm as the drop is moved through the focal plane. The third step is to align this new image and the original image to determine any displacement and thereby convert marked points and regions to the goniometer position.

For oscillation data collection we include an additional analysis for *in focus* features in a narrow region around the marked point to ensure that the marked point is on the rotation axis and stays in the beam during oscillation. Finally, we do also have to shift the drop position before starting data collection to correct for the optical distortion caused by the plastic and liquid. This is a plate-specific parameter and varies between 30 and 180 µm for the plates tested on the beamline so far.

### 5.4. Data processing and outputs via SynchWeb   

**Figure 6:**
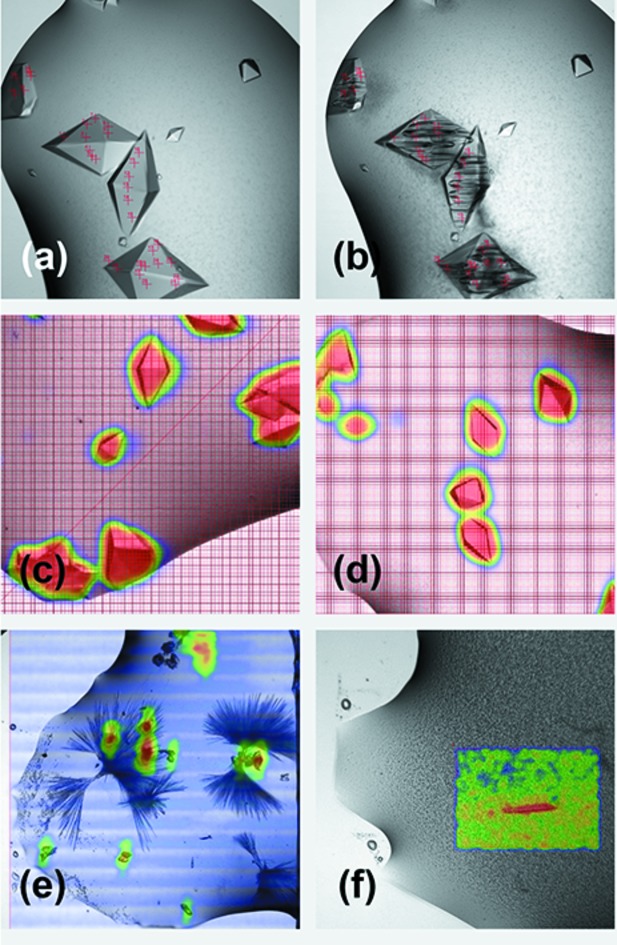
Examples of data collection outputs in SynchWeb. (*a*, *b*) Images before and after data collection. Note the extreme radiation damage observed in the areas marked for data collection. (*c*, *d*) Area scan over groups of crystals. The overlaid heat-maps show where the highest number of diffraction spots were recorded. Similar overlay for (*e*) an area with crystalline material and long needles and (*f*) precipitated protein obscuring a crystal.

When the requested diffraction data collections for a plate have been completed and are available for review and processing, the user is notified by email. At the moment, automatic data processing relies on existing pipelines used by other MX beamlines at DLS. For example, Xia2 (Winter *et al.*, 2013 ▸) image analysis is used to analyse grid scans and produce heat maps that can be displayed in SynchWeb [Figs. 6(*c*)–6(*f*) ▸]. Xia2 is also used to index, integrate and scale the individual oscillation datasets using *DIALS* (Winter *et al.*, 2018 ▸) (Table 3 ▸).

Due to the high volume of data and the complexity of the decisions needed to arrive at a final dataset, further developments in software are ongoing. They will eventually allow users to easily review hundreds of partial datasets across multiple plates. The outcome can either feed back into the crystallization process or piece together all the partial information to a complete dataset and structure determination.

## 6. Results   

The beamline user programme is still in its infancy. During the first two runs with limited user access, five user groups have used VMXi and around 30 plates have been loaded onto the beamline producing around 1500 datasets. The beamline is proving useful at both screening through crystallization conditions and for oscillation data collections.

As shown in Fig. 6(*a*) ▸, users are able to mark points of interest for the beamline to collect and, as seen in Fig. 6(*b*) ▸, once these data collections are executed, samples are usually destroyed. Nevertheless, as illustrated in Table 3 ▸, each individual data collection (typically spanning 60°) produces good statistics and these data collections can be easily merged in order to produce a complete dataset.

Users have also been able to screen through their samples and, by assessing the resulting heat maps in SynchWeb [Figs. 6(*c*) and 6(*d*) ▸], have found suitable conditions for data collection that are currently being tested further. One user group has been able to discard certain conditions due to the presence of salt crystals and others have been able to separate between two different crystal forms within the same condition as seen in Figs. 6(*e*) and 6(*f*) ▸.

## 7. Discussion   

The VMXi beamline is now providing dedicated *in situ* capabilities to the MX community. The improved microfocus optics with two monochromators options, a unique sample handling setup and 750 Hz data collection give this beamline unique capabilities that will enable cutting-edge science for some of the most challenging projects in structural biology. The automated and remote operation will improve the link between the work taking place in the laboratory and the resulting data quality, thereby speeding up the process from target selection to structural information. The high throughput and automated processing will make multi-crystal data collection routine and enable users to undertake new kinds of experiments on their samples that are not currently possible or are too complex.

With the increased availability of *in situ* beam time, the crystallization plate will now also serve as the sample holder. The current available plates and holders are likely to evolve. Improving the precision of drop location and the X-ray transparency are key developments required. Setting up crystallization between X-ray transparent sheets has already proven to greatly reduce optical artefacts and improve sample localization (Axford *et al.*, 2016 ▸) and will be available to users soon. This format is also well suited for data collection from membrane protein crystals grown in the lipidic cubic phase. We also suggest revisiting the free interface diffusion approach to crystallization, now that *in situ* data collection means crystals do not have to be recovered or subsequently be reproduced in a vapour diffusion setup.

The importance of structure-based drug design is clearly recognized in the commitment of all major pharmaceutical companies as a fully embedded process in the early stage of their drug discovery programs. The XChem facility based at DLS dedicated to fragment screening has been hugely successful operating with a cryo-crystallography approach, but is likely to benefit from an *in situ* approach for at least initial screening, something we will explore further in the coming year. The potential advantage would be to increase the chances of finding bound fragments by collecting diffraction data from more crystals in each drop *in situ* than are currently harvested for cryo-cooling. Sufficient high-quality data from multiple samples would help to assess fragment binding without the need for additional sample handling.

Further to *in situ* data collection, the unique properties of VMXi provide a platform for exploring synchrotron-based serial crystallography (Diederichs & Wang, 2017 ▸; Owen *et al.*, 2017 ▸). In collaboration with UK XFEL Hub, we are testing the most suitable types of experiments that can be added to the VMXi portfolio. For some projects, VMXi will provide the speed and flux required to generate serial crystallography data, while for other projects it can provide the required beam time to optimize sample handling and delivery in preparation for XFEL beam time. Once the requirements and scope are better defined, the beamline user program can be adapted to suit these requirements helping the user community make serial crystallography data collection more accessible.

## Figures and Tables

**Table 1:** Global beamline specifications

Beamline name	VMXi
Source	U23 undulator
Length	2 m
Period	23 mm
Minimum gap	5.5 mm
Diamond pre-DMM filters	Energy range
20 µm	10.0–12.5 keV
50 µm	12.5–16.6 keV
100 µm	16.5–21.5 keV
400 µm	21.5–25.0 keV
Double-multilayer monochromator (DMM)	
Lattice	Ru/B4C
*d*-spacing	2 and 2.4 nm
Band-pass (Δ*E*/*E*)	<5 × 10^−2^
Cooling	Water
Energy range	10–25 keV
Output flux at 13 keV	>1 × 10^14^ photons s^−1^
Double-crystal monochromator (DCM)	
Lattice	Si(111)
*d*-spacing	3.1475 Å
Band-pass (Δ*E*/*E*)	<10^−4^
Cooling	Liquid N_2_
Energy range	5–25 keV
Output flux at 13 keV	>2 × 10^12^ photons s^−1^
Pre-focusing horizontal focusing mirror (HFM)	
Distance from source	30.9 m
Length	1 m
Angle	2.7 mrad
Coating	Rh/Bare/Pt
Horizontal microfocusing mirror (HMFM)	
Distance from source	45 m
Length	1 m
Angle of incidence	3 mrad
Focal range at the sample	0–300 mm
Coating	Rh/Bare/Pt
Horizontal beam size at sample	5–30 µm
Vertical microfocusing mirror (VMFM)	
Distance from source	46 m
Active length	0.6 m
Angle of incidence	3 mrad
Focal range at the sample	0–300 mm
Coating	Rh/Bare/Pt
Vertical beam size at sample	2–30 µm
Available apertures diameters	10/20/200 µm

**Table 2:** Endstation equipment

Goniometry	
Main rotation axis	Vertical air bearing
Total oscillation range	−45° to +45°
Useful oscillation range at 60° s^−1^	−30° to +30°
Maximum rotation speed	90° s^−1^
Maximum rotation speed during data collection	60° s^−1^
Horizontal travel range	110 mm
Vertical travel range	80 mm
Maximum translation speed	100 mm s^−1^
Maximum translation speed during data collection	10 mm s^−1^
Tilt angle	15°

Sample shutter	
Maximum rotation speed	6000 rev min^−1^ or 36000° s^−1^
Shutter open/close maximum frequency	800 Hz
Opening time at full speed	50 µs

Photon counting detector	Eiger X 4M (Dectris)
Standard frame rate	500 Hz
Maximum frame rate	750 Hz
File format	Hdf5/Nexus
Pixel size	75 µm × 75 µm
Active surface (H × V)	155.2 mm × 162.5 mm
Total number of pixels (H × V)	2070 × 2167 = 4485690
Count rate (photons s^−1^ mm^−2^)	5 × 10^8^
Count rate (photons s^−1^ pixel^−1^)	1130000
Count rate (photons pixel^−1^ frame^−1^ at 750 Hz)	1500

Fluorescence detector	Vortex-60EX silicon drift X-ray detector

**Table 3:** Data collection statistics using the DMM beam (five thaumatin crystals from the same crystallization drop) Data collections wedges of 60° collected in 1 s each. All datasets were trimmed to only use the best wedge of data and resolution was cut to obtain complete data in all resolution shells. Data reprocessing of each wedge was undertaken using the available tools in SynchWeb. Merging of the final dataset was achieved using DIALS.

Dataset number in SynchWeb [see Fig. 5(*a*) ▸]	Dataset 1	Dataset 12	Dataset 24	Dataset 30	Dataset 35	All (2.2 Å cut-off)	All (2.0 Å cut-off)
Images collected	1–600	1–600	1–600	1–600	1–600	30000	30000
Images used for processing	100–600	1–450	200–600	1–250	200–400	18000	18000
Space group	*P*4_1_2_1_2	*P*4_1_2_1_2	*P*4_1_2_1_2	*P*4_1_2_1_2	*P*4_1_2_1_2	*P*4_1_2_1_2	*P*4_1_2_1_2
Unit-cell parameters (Å)	*a* = *b* = 58.7, *c* = 151.6	*a* = *b* = 58.57, *c* = 151.28	*a* = *b* = 58.54, *c* = 151.25	*a* = *b* = 58.54, *c* = 151.28	*a* = *b* = 58.62, *c* = 151.62	*a* = *b* = 58.6, *c* = 151.4	*a* = *b* = 58.6, *c* = 151.39
Resolution range (Å)	46.42–1.87 (1.92–1.87)	54.62–1.86 (1.91–1.86)	38.20–1.86 (1.91–1.86)	28.74–1.87 (1.92–1.87)	50.54–2.15 (2.21–2.15)	54.65–2.2 (2.26–2.2)	54.65–2.2 (2.26–2.2)
*R*_merge_ (all *I*+ and *I*−)	0.055 (0.139)	0.068 (0.173)	0.082 (0.131)	0.074 (0.369)	0.095 (0.462)	0.089 (0.170)	0.095 (0.312)
*R*_meas_ (all *I*+ and *I*−)	0.061 (0.197)	0.080 (0.173)	0.097 (0.186)	0.092 (0.522)	0.122 (0.653)	0.093 (0.187)	0.095 (0.376)
*R*_pim_ (all *I*+ and *I*−)	0.026 (0.139)	0.041 (0.173)	0.026 (0.131)	0.054 (0.369)	0.068 (0.453)	0.025 (0.074)	0.026 (0.204)
Total No. of reflections	44178 (97)	47524 (89)	35031 (55)	21362 (55)	16433 (447)	152069 (4513)	163393 (1901)
No. of unique reflections	12188 (84)	14435 (71)	11821 (50)	11294 (45)	8114 (396)	13793 (848)	16951 (750)
Mean *I*/σ(*I*)	17.2 (3.1)	11.9 (1.9)	7.3 (2.2)	8.1 (2.8)	4.8 (1.1)	20.5 (9.6)	17.9 (4.6)
Mn(I) half-set correlation CC(1/2)	0.997 (0.956)	0.993 (0.768)	0.990 (0.967)	0.986 (0.873)	0.994 (0.811)	0.993 (0.974)	0.993 (0.846)
Completeness (%)	53.9 (5.3)	62.5 (4.2)	52.0 (3.2)	49.8 (2.8)	54.5 (38.6)	97.4 (83.6)	97.4 (57.2)
Multiplicity	3.6 (1.2)	3.3 (1.3)	3.0 (1.1)	1.9 (1.2)	2.0 (1.1)	11.0 (5.3)	9.6 (2.5)
Mean (χ^2^)	0.91 (0.00)	0.97 (0.66)	0.94 (0.00)	0.85 (0.00)	0.97 (1.01)	0.94 (0.82)	0.93 (0.75)
